# The spatial non-stationary effect of urban landscape pattern on urban waterlogging: a case study of Shenzhen City

**DOI:** 10.1038/s41598-020-64113-1

**Published:** 2020-04-30

**Authors:** Jiansheng Wu, Wei Sha, Puhua Zhang, Zhenyu Wang

**Affiliations:** 1 Key Laboratory for Urban Habitat Environmental Science and Technology, School of Urban Planning and Design, Peking University, Shenzhen, 518055 PR China; 2 Laboratory for Earth Surface Processes, Ministry of Education, College of Urban and Environmental Sciences, Peking University, Beijing, 100871 PR China

**Keywords:** Environmental impact, Urban ecology

## Abstract

The problem of urban waterlogging has consistently affected areas of southern China, and has generated widespread concerns among the public and professionals. The geographically weighted regression model (GWR) is widely used to reflect the spatial non-stationarity of parameters in different locations, with the relationship between variables able to change with spatial position. In this research, Shenzhen City, which has a serious waterlogging problem, was used as a case study. Several key results were obtained. (1) The spatial autocorrelation of flood spot density in Shenzhen was significant at the 5% level, but because the Z value was not large it was not very obvious. (2) The degree of impact on flood disasters from large to small was: Built up_ DIVISION > SHDI > Built up_ COHESION > CONTAG > Built up_ LPI. (3) The degree of waterlogging disasters in higher altitude regions was less affected by the landscape pattern. The results of this study highlight the important role of the landscape pattern on waterlogging disasters and also indicate the different impacts of different regional landscape patterns on waterlogging disasters, which provides useful information for planning the landscape pattern and controlling waterlogging.

## Introduction

In recent years, due to the rapid urbanization experienced in China, the nature of the underlying surface of urban areas throughout the country has undergone significant and dramatic changes, resulting in frequent urban disasters and huge losses of life and property^1^, with further changes to the hydrological characteristics of urban rainwater systems^2^. Some studies have established multivariate regression models based on a geographic information system (GIS) to determine the factors influencing urban waterlogging^3^. Other studies have investigated the characteristics of urban road traffic congestion under the influence of heavy rain^4^. Chinese researchers evaluated the risk of urban storm and flood disasters in the context of land use changes in the Maozhou River Basin, Shenzhen^5,6^ analyzed the spatiotemporal pattern of thyroid cancer (TC) and considered the relevant environmental risk factors in Hangzhou (HZ). Chen, *et al*.^7^ studied the impact of land use and population density on seasonal surface water quality using a modified geographically weighted regression (GWR) model^8,9^. Studies of the water transport capacity and rainwater utilization in urban drainage systems outside of China are relatively mature^10,11^. Many researchers have used the ArcGIS software, remote sensing (RS), and the global positioning system (GPS) at micro^12^ and macro^13^ scales to study the problems of water accumulation and flood control in cities. These studies have mainly focused on traditional water conservancy and drainage pipe networks, and other artificial engineering facilities. The natural and artificial landscapes have different ecological patterns, such as the urban environment, non-point source pollution, and the imbalance of aquatic ecosystems^14–16^. In recent years, many researchers have studied the causes of urban waterlogging and the relationship between urban sprawl^17–19^, land use^20–23^ and human activity^24–28^ from the perspective of ecology. In addition, there have been some studies that have investigated sponge-like urban reconstruction and the urban-inundation simulation method^29–31^.

Due to its rapid development, in recent years RS technology has become an important tool in landscape ecology research. Using RS images, various landscape pattern indexes can be calculated to quantitatively describe the size, density, edge, shape, and spatial distribution of landscape patches. This enables the characteristics and laws of landscape pattern evolution to be analyzed at different scales^32–37^.

Compared with the OLS model, the GWR model has the following advantages. First, when processing spatial data, the parameter estimation and statistical testing of the model are more significant than in the OLS model, and have smaller residuals. Second, the spatial unit of each sample in the GWR model corresponds to a coefficient value, and therefore the model results can reflect the local situation more accurately than in the OLS model. The GWR model can restore the specific characteristics of the relationship between variables ignored by the OLS model. Finally, the parameter estimation of the model can be spatially expressed through ArcGIS, which is convenient for further constructing the geographic model and exploring spatial variability and spatial law^38^.

Many researchers have used the GWR model rather than the OLS model to analyze the spatial relationship of different factors. Zhou, *et al*.^39^ used the GWR model to analyze the cause of haze pollution in China and found that its estimates were better than those of the OLS estimate, with an improvement in the R^2^ value from 0.20 to 0.75. Song, *et al*.^40^ created a land use regression model for NO_2_ and NO and then use OLS and GWR model to estimate the effects of urban land-use configuration on NO_2_ and NO concentrations and found that the GWR model was more accurate than OLS model, with increases of 29.3% and 6.9%, respectively. Using the GWR model, Frutos, *et al*.^41^ found that low atmospheric pressure may increase depression and suicide by inducing hypoxia, while previous studies had not evaluated the geographic variation of this relationship across the United States. The present study used the GWR model to study the spatial non-stationarity between variables and reached various conclusions. Fotheringham, *et al*.^42^ proposed a model based on GWR, which is a modeling technique that effectively deals with spatial non-stationary phenomena through regression analysis. By introducing the spatial position of the data into the regression coefficients, the non-parametric estimation method can be used to provide a local estimator of the function in each geographic location. The regression relationship is mainly explored and analyzed according to the variation of the regression coefficient at each geographical location, with the change of space. Many researchers have used the GWR model to analyze the relationships among factors with spatially distinct characteristics^6,43–46^. With regard to studies of spatial non-stationarity, most scholars have used the GWR model in the socio-economic field^47–51^.

The aim of this paper is to explore the spatial non-stationary nature of the GWR model and study the relationship between urban landscape pattern and urban waterlogging in densely populated areas and urban built-up areas. Besides, Shenzhen is the city with the fastest urbanization in China, with a small area and a large population, and the highest population density and construction density per unit area, which is a typical case to realize the aim of the study. Compared with the previous paper, this paper not only discusses the relationship between urban landscape pattern and waterlogging degree, but also studies the spatial non-stationarity of GWR Model, which reaches the goal of killing two birds with one stone.

## Results

### Benchmark regression analysis

In order to make the experimental results more credible, before using the GWR regression analysis, a global OLS test is performed first.

Tables 1 and 2 show that basic OLS regression. In order to solve the multicollinearity problem of data, according to the VIF of variables, the collinearity factors are removed by using the backward stepwise method. lnPrecipitation is removed in columns (1) and (2) step by step.

**Table 1:** OLS test results (4 land use classes).

Variables	(1)	(2)	(3)	(4)	(5)
	lnDWS	lnDWS	lnDWS	lnDWS	lnDWS
lnLPI	0.1285	0.14723	0.0917*	0.1126	0.1322
	(0.1467)	(0.2456)	(0.0645)	(0.3125)	(0.2472)
lnCOHESION	0.0475	0.0342*	0.0418	0.0385	0.0521*
	(0.1376)	(0.0203)	(0.0531)	(0.1312)	(0.0312)
lnDIVISION			−2.0186	−3.0331	−3.0274
			(0.0254)	(0.0421)	(0.0384)
lnCONTAG				0.1632	0.1462
				(0.2315)	(0.1971)
lnSHDI					−1.2156
					(0.2486)
lnPrecipitation		2.5317*	1.9874**	2.8716*	3.1762***
		(1.8434)	(0.8434)	(2.1273)	(0.1972)
Constant	15.7541*	11.0589*	10.2675*	18.3247	13.1526**
	(12.3816)	(9.1847)	(8.6541)	(20.3987)	(6.8712)
Radj^2^	0.058	0.134	0.157	0.166	0.175

Standard errors in parentheses.

***p < 0.01, **p < 0.05, *p < 0.1.

Density of waterlogged sites = DWS.

**Table 2:** OLS test results (16 land use classes).

Variables	(1)	(2)	(3)	(4)	(5)
	lnDWS	lnDWS	lnDWS	lnDWS	lnDWS
lnLPI	0.0973	0.1137*	0.0816	0.1573	0.1727
	(0.1318)	(0.0774)	(0.1345)	(0.2517)	(0.2119)
lnCOHESION	0.0714	0.0578	0.0813	0.0687	0.0952
	(0.1784)	(0.0715)	(0.0934)	(0.0881)	(0.1748)
lnDIVISION			−3.0186	−2.0331*	−1.0274*
			(6.7154)	(1.8921)	(0.8356)
lnCONTAG				0.1128	0.1276*
				(0.2315)	(0.0817)
lnSHDI					−2.2156
					(0.2486)
lnPrecipitation		4.1324**	1.7623**	1.7687*	4.8786**
		(1.8434)	(0.9817)	(2.8136)	(2.3365)
Constant	20.3184*	18.7565*	16.8673*	15.1341	17.8616***
	(14.9176)	(14.3845)	(12.6732)	(17.5361)	(3.2984)
Radj^2^	0.138	0.204	0.213	0.224	0.231

Standard errors in parentheses.

***p < 0.01, **p < 0.05, *p < 0.1.

Density of waterlogged sites = DWS.

Additionally, most of the variables have a positive impact on Density of waterlogged sites; only DIVISION and SHDI are negative. We also find the following. (1) The larger the area of construction land is, the more likely the urban waterlogging will occur. (2) The denser the urban landscape, the more prone to waterlogging. (3) The more complex the landscape of building land is, the less likely it is to be flooded. (4) The coefficient of precipitation is large, which has a significant impact on urban waterlogging. (5) Considering the overall indicators, there was no significant difference in all regression factors of the all variables in 4 land use classes and 16 land use classes, and the adjusted R^2^ are approximately 0.15 in 4 land use classes and 0.20 in 16 land use classes, and the fitting degree is not high.

Since rainfall has a great influence on the model, this factor is eliminated in the GWR Model.

### Autocorrelation analysis of urban waterlogging in Shenzhen City

**Figure 1:**
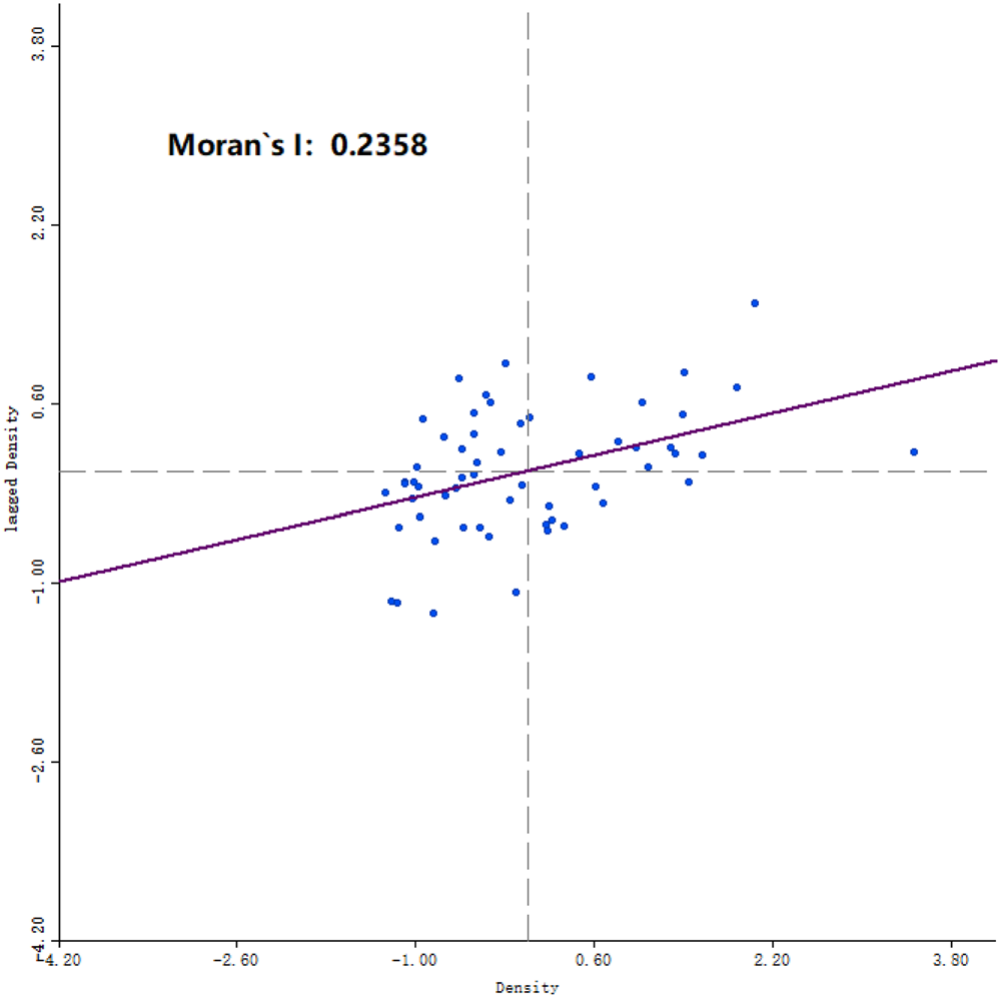
The scatter plot of Moran’s I values for waterlogged site density in Shenzhen City.

Based on the global Moran’s I theory, 56 small watersheds in Shenzhen where waterlogging occurred were used as spatial units, and the average density of waterlogged sites in each small watershed was used as the observation value. Using Geoda software, a distance weighting matrix (DWM) was selected to analyze the spatial autocorrelation of waterlogged sites. It can be seen from Fig. 1 that the global Moran’s I index was 0.236. After the significance test, the normalized Z value was 2.913335 (>1.96), indicating that the spatial autocorrelation of waterlogged site density in Shenzhen was significant at the 5% significance level. However, because the Z value was not large, the spatial autocorrelation was not very obvious.

**Figure 2:**
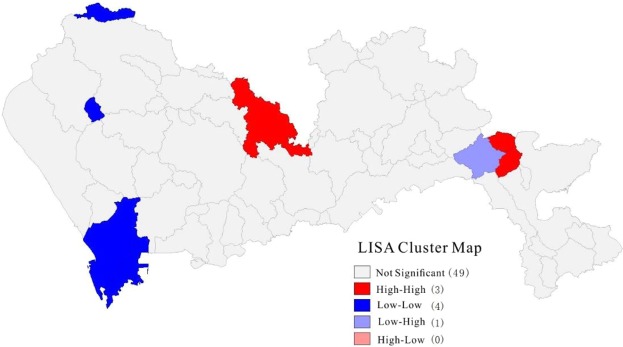
An aggregated local indicators of spatial association (LISA) map of waterlogged site density in Shenzhen City.

It can be seen from the LISA cluster map (Fig. 2) that there was a wide range in the spatial distribution of the density of waterlogged sites in Shenzhen. Nanshan District, Baoan District, and Guangming New District in the west were low-low accumulation areas, indicating that the resistance to waterlogging disasters in this and surrounding areas was strong. A small part of Longhua District and Dapeng New District were high-high accumulation areas, indicating that these and surrounding areas were prone to waterlogging disasters. Small parts of Dapeng New Area were low-high accumulation areas, indicating that these areas with a low waterlogged site density were surrounded by waterlogging-prone areas, which represented a low to high transitional area of waterlogging. Overall, there was a weak spatial agglomeration of the density of waterlogged sites in Shenzhen. The only variations occurred in local areas, reflecting the non-stationary characteristics of the spatial distribution of the degree of waterlogging disasters.

### GWR model establishment and test results

Due to the differences in the distribution of urban landscape patterns, the spatial distribution of waterlogged sites in the urban area was characterized by non-stationary features. Therefore, the land use types in Shenzhen were divided into four and 16 categories to calculate the landscape pattern index. The two groups were established and a total of 10 landscape pattern indexes in each group were obtained and used as independent variables. At the same time, due to the influence of daily rainfall and topography, the GWR4.0 software was used to construct the GWR model with the average slope and average rainfall as independent variables.

It can be seen from Table 3 that when the land use was divided into four types, the Built up _DIVISION had the greatest influence on the density of waterlogged sites, with a negative correlation, i.e., the more broken the patches of built-up land, the more serious the potential waterlogging disaster. The SHDI also had a significant negative correlation with the density of waterlogged sites, which means that the more complex the patch of built-up land, the more serious the potential waterlogging disaster. Of the three landscape pattern indexes that had a positive correlation with the degree of waterlogging disaster, the most influential was the Built up _COHESION, i.e., the greater the potential for the built-up land to experience a waterlogging disaster.

**Table 3:** Regression coefficients for the relationships between the density of waterlogged sites and landscape pattern indexes from the geographically weighted regression (GWR) model.

	Variable	Average	Minimum	Lower quartile	Median	Upper quartile	Maximum	AIC	R^2^	R_adj_^2^	F	SD	t
4 class	Built up_LPI	3.625	3.317	3.507	3.646	3.881	4.085	658.56	0.443	0.346	2.712	0.818	4.208
	Built up_COHESION	93.705	42.330	58.724	101.123	133.691	150.749	658.88	0.482	0.352	2.570	18.60	3.758
	Built up_DIVISION	−356.89	−390.695	−378.674	−363.753	−348.88	−323.99	657.34	0.455	0.361	2.525	75.99	−4.51
	CONTAG	5.835	5.533	5.870	5.975	6.056	6.157	657.24	0.446	0.360	1.978	1.159	4.903
	SHDI	−270.72	−288.880	−282.301	−275.992	−270.99	−253.64	663.72	0.385	0.283	2.182	67.37	−3.79
16 class	Built up_LPI	5.726	1.138	3.599	5.724	8.468	9.743	657.27	0.521	0.371	3.290	1.526	2.607
	Built up_COHESION	36.313	25.345	32.589	37.927	41.976	45.374	650.27	0.558	0.443	2.258	6.818	5.195
	Built up_DIVISION	−889.47	−1929.27	−1435.97	−809.773	−432.21	−148.97	663.90	0.460	0.291	3.031	261.8	−1.44
	CONTAG	7.855	4.748	6.451	7.888	9.742	11.308	671.28	0.336	0.186	2.217	3.274	1.903
	SHDI	−197.51	−266.164	−242.705	−201.611	−169.15	−123.84	669.85	0.354	0.207	2.311	73.66	−2.13

When the land use was divided into 16 types, the Built up_ DIVISION and SHDI had a significant negative correlation with the density of waterlogged sites. The greater the value of Built up_ LPI, CONTAG, and Built up_ COHESION, the greater the potential for a waterlogging disaster.

Regardless of whether the land use type was divided into four or 16 categories, the impact on waterlogging disasters followed the order of Built up_ DIVISION > SHDI > Built up_ COHESION > CONTAG > Built up_ LPI. This was mainly because the values of Built up_ DIVISION and SHDI were smaller than those of the other indexes, and therefore the regression coefficient values were large. In addition, it can be seen from Table 4 that the regression coefficient of each landscape pattern index had an obvious non-stationarity in Shenzhen. The ability to determine this is the largest advantage of using the GWR model in spatial data analysis.

**Table 4:** Overview of data used in this study.

Data type	Properties	Source
Waterlogging point data of Shenzhen during on rainstorm period on May 11, 2014	A total of 278 points	Shenzhen Flood Control and Drought Prevention and Wind Control Headquarters
Land Use Data 2013 of Shenzhen	30 × 30(m)	Shenzhen Government
DEM Data of Shenzhen	30 × 30(m)	Geospatial Data Cloud
Daily Rainfall Data for Shenzhen, May 11, 2014	Statistical data, recorded by 50 meteorological monitoring stations	Shenzhen Meteorological Bureau website

### Non-stationarity verification of the GWR model

**Figure 3:**
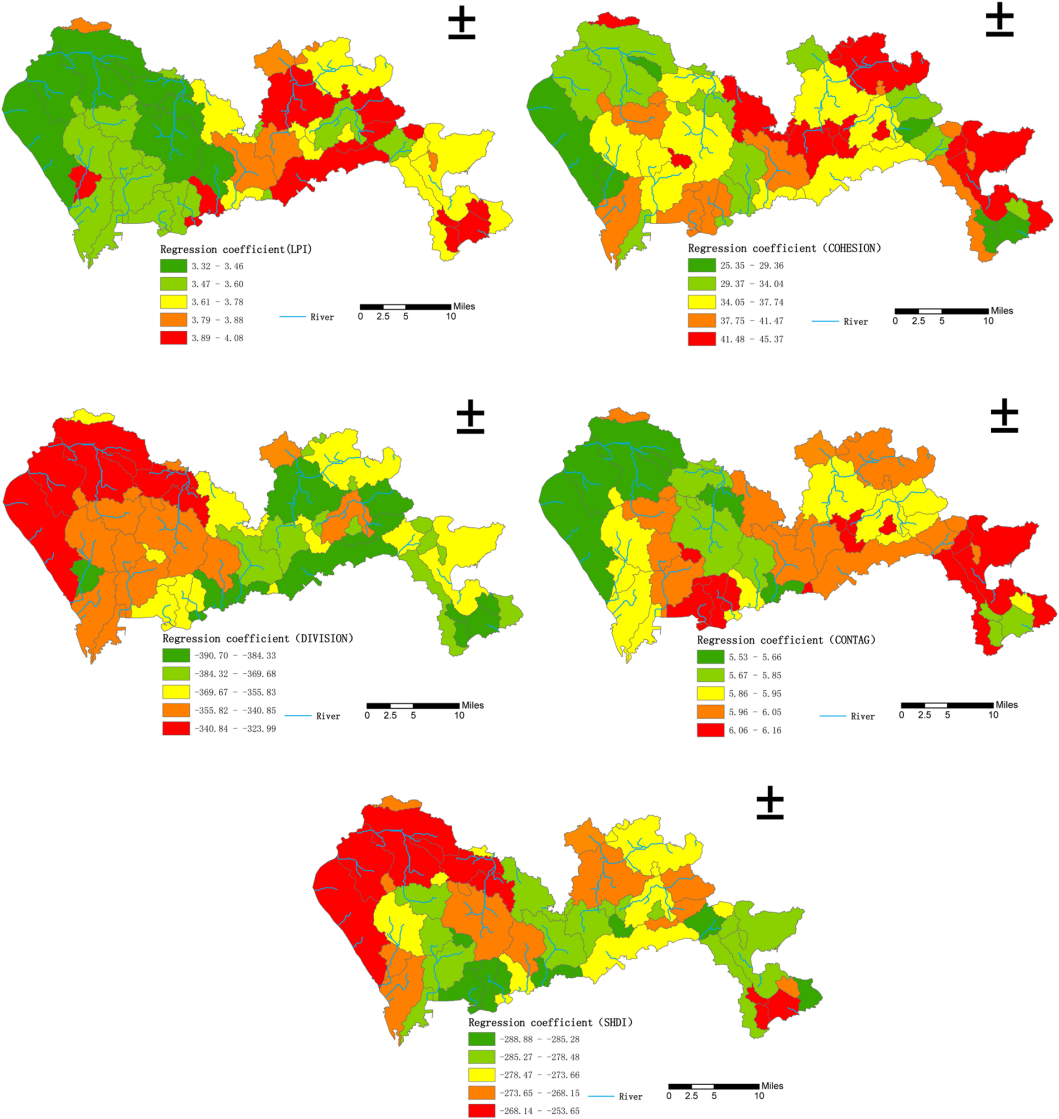
Distribution of regression coefficients for the relationships between the density of waterlogged sites and landscape pattern indexes (4 land use classes) in Shenzhen.

As shown in Fig. 3, when the land use types were divided into four categories, the regression coefficient of the landscape pattern indexes and the density of waterlogged sites showed an obvious spatial non-stationarity. For Built up_ LPI, Built up_ COHESION, and CONTAG, low regression coefficient values were mainly distributed in Baoan District, Guangming New District, and Longhua District in the northwest of Shenzhen, followed by Nanshan District and Futian District in the southwest and Pingshan District in the central and eastern regions. These areas are the main population centers in Shenzhen, and the land use types are mainly large areas of built-up land and fragmented green spaces, and therefore the Built up_ LPI, Built up_ COHESION, and CONTAG values were high, leading to small regression coefficients. In Longgang District, Yantian District, and Dapeng New District in the northeast, south central, and southeast of Shenzhen, due to the complex land use type, the Built up_ LPI, Built up_ COHESION, and CONTAG values were low, resulting in a large regression coefficient.

For Built up_ DIVISION and SHDI, low regression coefficients were mainly distributed in Futian District, Luohu District, and Yantian District in the south-central part of Shenzhen, followed by part of the Dapeng New District in the southeast and part of Longgang District in the northeast. These areas are located in the old communities of Shenzhen, where urban planning in the early years of the city’s development was inadequate. The land use type is mainly fragmented built-up land and small patches of green land, and therefore the Built up_ DIVISION index and SHDI index values were high, resulting in small regression coefficients. Baoan District, Guangming New District, and Longhua District, located in the western part of Shenzhen City, had a relatively large regression coefficient due to the relatively simple land use type and large patches of built-up land, resulting in low Built up_ DIVISION index and SHDI index values.

Overall, under the rough classification accuracy of land use, the regression coefficients of Built up_ LPI, Built up_ COHESION, and CONTAG had a spatial distribution pattern of west low-east high. In contrast, the regression coefficients of Built up_ DIVISION and SHDI had a spatial distribution pattern of west high-east low.

**Figure 4:**
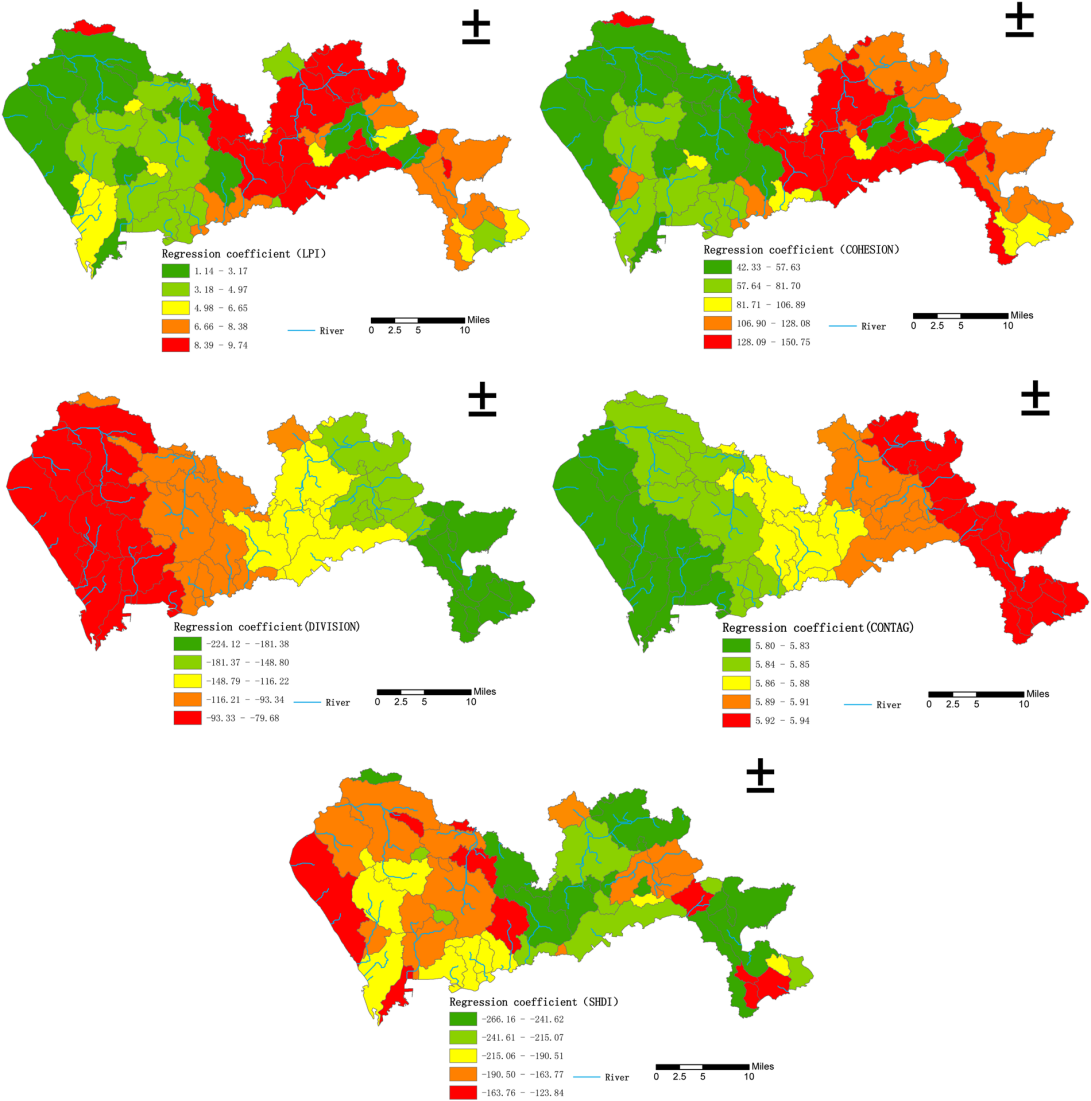
Distribution of regression coefficients for the relationships between the density of waterlogged sites and landscape pattern indexes (16 land use classes) in Shenzhen.

When the land use type was divided into 16 categories, the regression coefficients of the landscape pattern index values and the density of waterlogged sites also displayed an obvious spatial non-stationarity. For the Built up_ LPI, Built up_ COHESION, and CONTAG indexes, the spatial distribution of the regression coefficients was similar to that when the land use types were classified into four categories, with a spatial distribution pattern of west low-east high. Additionally, the regression coefficient for CONTAG had significant layering characteristics. For the Built up_ DIVISION and the Built up_ SHDI the spatial distribution of the regression coefficients was similar to that when the land use type was divided into four categories, with a spatial distribution pattern of west high-east low. Additionally, the regression coefficient for Built up_ DIVISION displayed clear layering characteristics, as shown in Fig. 4.

Comparing the landscape pattern indexes calculated by the GWR model established by dividing the land use type into four or 16 categories, it was found that under the rough classification, the regression coefficient of CONTAG changed slightly but the regression coefficient for Built up_ DIVISION changed substantially. The regression coefficients corresponding to the various landscape pattern indexes under the fine classification displayed greater spatial fluctuations than under the rough classification. The reason for this may be that after the land use types were subdivided, the differences in the various landscape pattern indexes were more obvious, as a result, the variation in the range of regression coefficients increased accordingly.

Because the regression coefficient distribution map displayed by Arc-GIS could not show the degree of each area affected by altitude, a spatial three-dimensional (3-D) scatter plot was produced using the Origin 9.0 software to represent the relationship between the landscape pattern indexes and the impact of waterlogging disasters at different latitudes, longitudes, and altitudes. Because the regression coefficients of the Built up _ DIVISION and SHDI were both negative, for the convenience of display in the 3-D scatter plot, the absolute values of the two indexes were selected.

**Figure 5:**
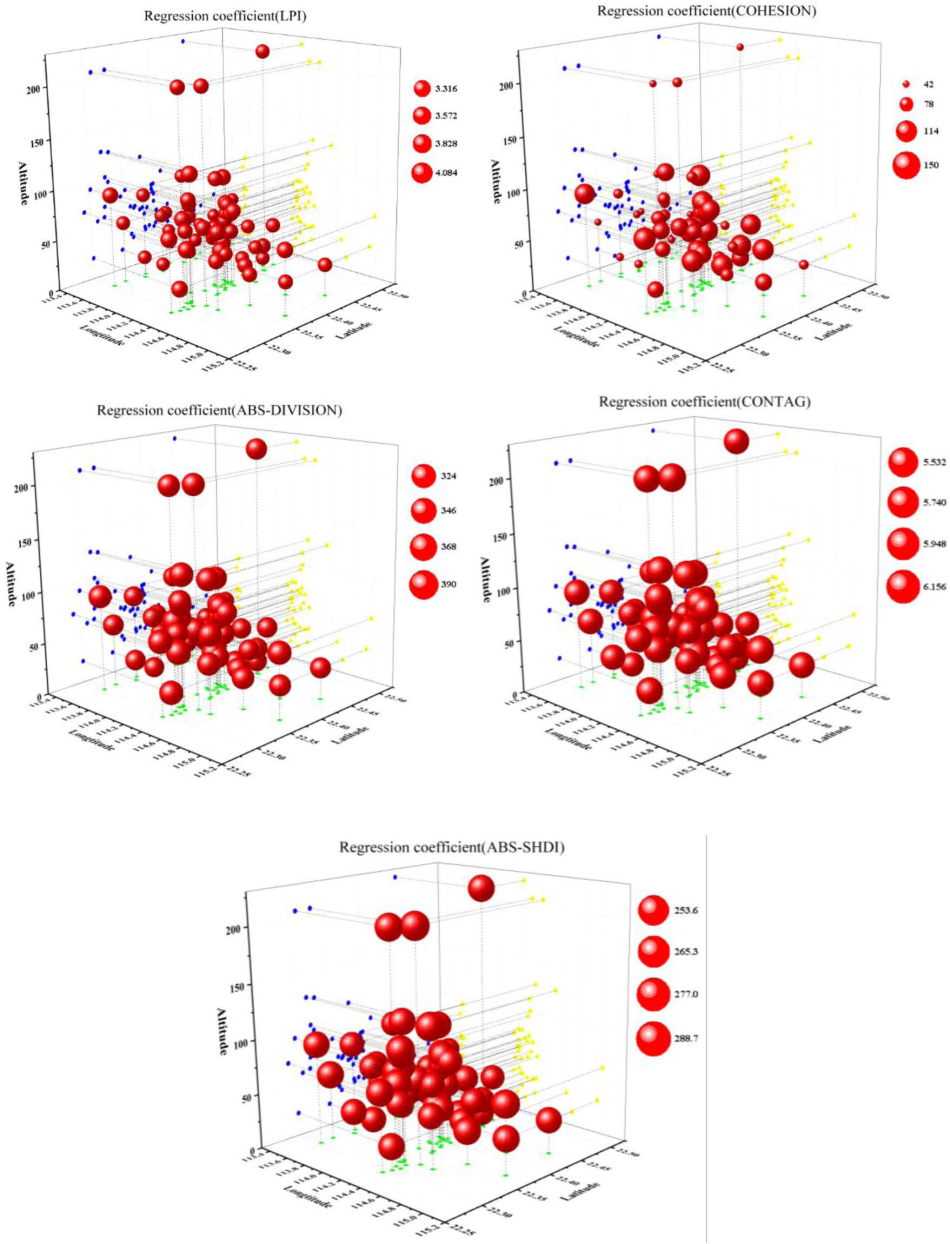
Three-dimensional 3-D scatter plot of regression coefficients for the relationships between the density of waterlogged sites and landscape pattern indexes (four land use classes) in Shenzhen.

As shown in Fig. 5, in the GWR model constructed by dividing the land use type into four categories, there was little spatial difference in the regression coefficient values and the values for Built up_ LPI and Built up_ COHESION when the altitude exceeded 100 m were significantly less than the regression coefficient values below 100 m. This indicates that the degree of damage from waterlogging disasters at high altitudes was less affected by the landscape pattern than at low altitudes. The regression coefficient of the other three landscape pattern indexes did not change significantly on the vertical scale.

**Figure 6:**
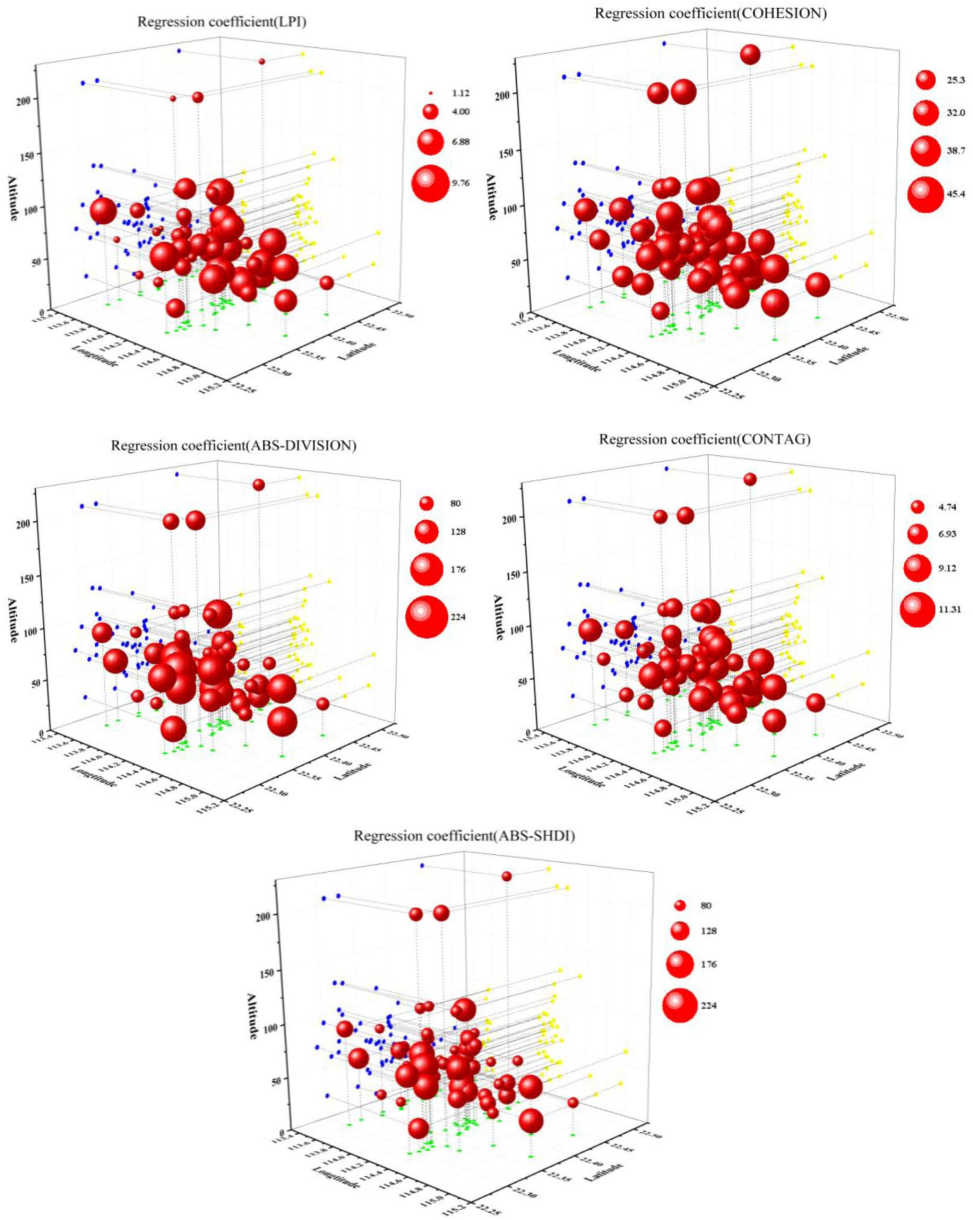
Three-dimensional 3-D scatter plot of regression coefficients for the relationships between the density of waterlogged sites and landscape pattern indexes (16 land use classes) in Shenzhen.

As shown in Fig. 6, in the GWR model constructed by dividing the land use type into 16 categories, the regression coefficients were significantly different in space. For Built up_ LPI, Built up_ COHESION, and Built up_ CONTAG the regression coefficients below 100 m were significantly smaller than the regression coefficients when the altitude was above 100 m. This indicates that the degree of damage from waterlogging disasters at high altitudes was less affected by the landscape pattern than at low altitudes. For the regression coefficients of the other two landscape pattern indexes, there was no significant change in the vertical scale.

## Discussion

Most previous studies focused on the impact of landscape pattern on watershed hydrological processes and urban waterlogging disasters^52^. The present study used the Shenzhen “5.11 rainstorm” in 2014 as a case study. Based on the actual distribution of all recorded waterlogged sites, a spatial autocorrelation analysis, GWR model, and other statistical methods were used to determine the urban landscape pattern (including type and landscape level), which had a spatial non-stationary impact on urban waterlogging disasters.

The results showed that human factors, such as land use type and landscape pattern, had a significant influence on urban waterlogging problems, with significant impacts in various urban areas. The results can be used to guide urban landscape pattern planning and optimization, and the construction of ‘sponge cities’. This further indicated that the spatial distribution of waterlogging disasters was related to the spatial heterogeneity of the landscape pattern, which provided reliable theoretical support for the subsequent establishment of the GWR model between the waterlogged site density and the landscape pattern index. There were several key implications for city managers. (1) Minimize the endless connection of large-scale built-up land, reduce the degree of accumulation of built-up land by means of green spaces or water bodies, and make their spatial distribution more discreet. (2) Waterlogging disasters in eastern Shenzhen were intensified by the landscape pattern. This area of the city is the location of many new districts, and therefore the government should consider the rationality of spatial pattern when planning. (3) The existing urban green space should be protected and integratedwhile maximizing the urban green area. Different green land sub-categories, such as forest and grassland can be treated differently, with an emphasis on increasing the spatial concentration of forests and gardens. (4) The terrain will have some impact on the degree of waterlogging disasters, with high-lying areas being less affected by the landscape pattern, so the low-lying area of the city needs reasonable planning to avoid waterlogging to the maximum extent.

There are also some limitations need to be addressed in future studies. Firstly, due to data availabilty, this study mainly focused on densely populated and urban built-up areas, but paid liitle attention to natural areas. Also, this study considered only Shenzhen, with no data from other cities, for which the results may therefore be very specific to the case study area. In addition, the spatial distribution of waterlogged sites was not very even, which may impact the results. GWR model is a linear regression model, but it only involves linear interpolation and has some limitations. Therefore, further model improvement and optimization should be explored in future research^53^. Landscape pattern index can only represent some characteristics of landscape, which also leads to the same landscape pattern index for two different urban landscapes.

Because of the difficulty of data acquisition, only waterlogging events following the “5.11 rainstorm” in 2014 were analyzed. If data for waterlogged sites corresponding to different storm events can be acquired in the future, the spatial non-stationary nature of the landscape pattern on waterlogging disasters under different storm intensities could be determined. This data could then be compared with the results of the present study to determine their timeliness and credibility. As more attention is given to disasters caused by heavy rain, the city’s disaster prevention and mitigation measures will improve, and political factors, such as urban management, will gradually become an important factor affecting urban waterlogging disasters. The quantification of this process will also be a focus of future studies of urban flooding.

## Conclusions

This study considered the spatial non-stationary nature of the GWR model by analyzing the relationship between landscape patterns and urban waterlogging disasters. First, it was found that the spatial autocorrelation of the density of flooded sites in Shenzhen was significant at the 5% significance level, but because the Z value was not large, spatial autocorrelation was not very obvious. Second, when the land use types were divided into four and 16 categories, the Built up_ DIVISION, SHDI, and density of waterlogged sites were negatively correlated, with the density of waterlogged sites, while the Built up_ LPI, CONTAG, and Built up_ COHESION were positively correlated with the density of waterlogged sites. Among the various landscape pattern indexes, the degree of influence on waterlogging disasters followed the order of: Built up_ DIVISION > SHDI > Built up_ COHESION > CONTAG > Built up_ LPI. Third, the regression coefficients of Built up_ LPI, Built up_ COHESION, and CONTAG had a spatial distribution pattern of west low-east high; while the regression coefficients of Built up_ DIVISION and SHDI had a spatial distribution pattern of west high-east low. Finally, the regression coefficient of Built up_ LPI and Built up_ COHESION was significantly less than the regression coefficient below 100 m when the altitude was higher than 100 m, indicating that the severity of waterlogging disasters in the higher altitudes was less affected by the landscape pattern. However, built up_ DIVISION and SHDI did not change significantly with altitude.

The outcomes of this study provided valuable reference information for the effective and rational use of land in Shenzhen city to avoid the occurrence of urban waterlogging, especially for the low altitude and densely populated areas. Because Shenzhen is a typical coastal developed area in China, which has many similarities with most coastal developed cities in China, the research results of this paper also have some reference value to other regions. The spatial nonstationarity of GWR Model is also proved in this study, and it can also have a good effect in the study of urban structure and urban waterlogging.

## Materials and methods

### Study area and data sources

#### Study area

**Figure 7:**
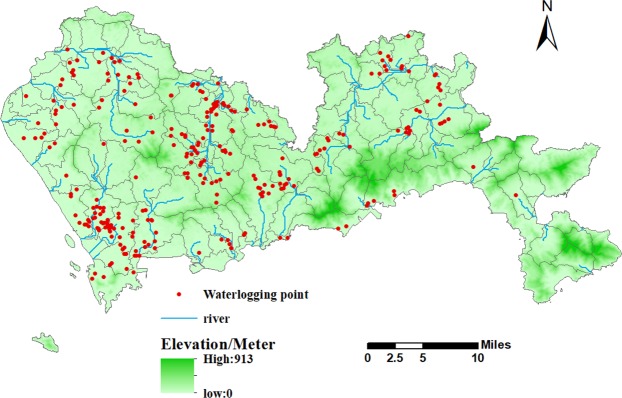
Distribution of waterlogging points and the Shenzhen sub-watershed.

Shenzhen City in Guangdong Province was used as a case study. Shenzhen is located in the southeast coastal area of China, in the south of Guangdong Province, on the east bank of the Pearl River Estuary. It is close to Hong Kong, and is one of the fastest growing and most developed regions in China. The city has nine administrative districts and one new district, with a total area of 2020 km^2^, and is located at 113°46′–114°37′E, 22°27′–22°52′N. Shenzhen is located in the subtropical maritime monsoon climate zone, and its climate is humid, rainy, warm, and frost- and ice-free throughout the year. It generally has long summers and short spring, autumn, and winter seasons. Due to the high average temperature and high humidity throughout the year, precipitation amounts are large, with an average annual precipitation of 1837 mm. However, due to the uneven terrain in Shenzhen and the presence of mountains in the city, the spatial and temporal distribution of precipitation is uneven. Precipitation is mainly concentrated in the April to September period of each year, which accounts for about 85% of annual precipitation. On a spatial scale, precipitation is concentrated in the southeastern part of the city, while in the northwest there is less rainfall. From east to west, there is a clear downward trend in rainfall. The spatial and temporal differences in the precipitation distribution have resulted in very serious urban waterlogging problems in Shenzhen and there is a need for better management and planning by the relevant departments. On May 11, 2014, Shenzhen suffered heavy precipitation, which led directly to serious levels of water accumulation in 150 roads across the city. More than 5,000 buses were forced to stop operating, more than 20 communities were affected, and more than 2,000 vehicles were flooded, resulting in huge economic losses and seriously affecting the movement and daily lives of urban residents. The official statistics provided by the Shenzhen Flood Control and Drought Prevention and Wind Control Headquarters showed that during the rainstorm, there were 278 locations where waterlogging occurred within the city (Fig. 7). Due to the wide extent and severe impact of the torrential rainstorm, this study investigated the spatial non-stationarity of Shenzhen’s waterlogging problem through a case study of landscape patterns at different classification levels.

Urban waterlogging is a systemic problem. The water cycle process produces distinct geographical basins, within which the problem of destruction due to waterlogging cannot be analyzed only from a certain point, but rather needs to be studied from the basin perspective. Based on digital elevation model (DEM) data, this study extracted the hydrological elements using the “Eight-Vector Method” (D8) to obtain a watershed division map of Shenzhen (Fig. 1), with a small watershed as the research unit.

#### Data sources

The torrential rain data used in this study were obtained from measurements made during the “5.11 rainstorm” in Shenzhen in 2014, with data extracted for 278 locations where waterlogging occurred during the rainstorm. Using ArcGIS10.1 (https://www.esri.com/zh-cn/arcgis/products) software to vectorize the data, a spatial dataset of the rainstorm was created. The dataset was superimposed with a watershed partitioning map, and 56 small watersheds containing waterlogged areas were extracted. The density of waterlogged points in each basin was calculated (waterlogging point density = number of waterlogging points in the basin/basin area, units: Per km^2^), and was used to characterize the extent of waterlogging in each small watershed. Previous studies have shown that urban waterlogging is mainly affected by natural factors such as meteorological conditions, topography, and terrain, and human factors such as land use and drainage facilities^54,55^. This study first classified land use types, obtained related variables, and then calculated the values of landscape pattern indexes (including land use type and landscape level indexes) using Fragstats software. These values were then combined with other variables known to influence waterlogging, including daily rainfall. The GWR model was used to explore the impact of the urban landscape pattern on the non-stationarity of urban waterlogging. All data used in the study are shown in Table 4.

### Land use-land cover(LULC) data

**Figure 8:**
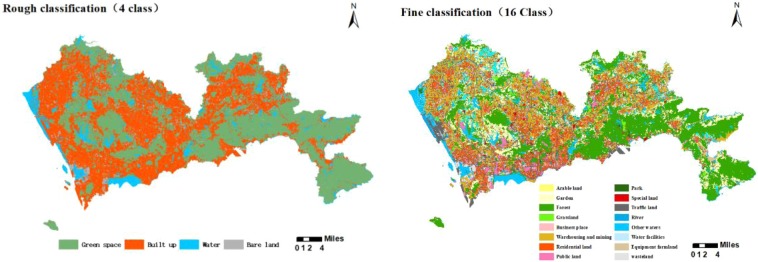
The classification of land use.

The original land use data was divided into 12 primary-classes and 56 secondary-classes based on the *land use classification standard (GB/T21010-2007)*. The data was reclassified according to the aims of the research, and finally a land use classification with rough and fine classification accuracy was obtained (Fig. 8). Then the landscape pattern indexes are calculated as independent variables in the model.

### Landscape pattern index

The landscape pattern index can concentrate the landscape pattern information, reflect the spatial composition and structural characteristics of the landscape, and is easy to calculate, and it is therefore widely used to measure the landscape pattern. The landscape pattern index was used to characterize the landscape pattern of the basin in this study. Taking the features of each landscape pattern index into account and based on the principle of statistical independence^56,57^, together with reference to existing research^58^, five indexes were selected, including three land use type indexes: largest patch index (LPI), patch cohesion index (COHESION), and landscape division index (DIVISION); and two landscape indexes: contagion (CONTAG) and Shannon’s diversity index (SHDI). A description of each index is shown in Table 5, and the calculation process was completed in Fragstats 4.2 software.

**Table 5:** Overview of landscape indices used in this study.

Scale	Index	Unit	Range	Representational meaning
Type level	(LPI)	%	0 < LPI ≤ 100	Representing the dominance of landscape types.
	(COHESION)	None	0 < COHESION < 100	Reflecting the degree of patch accumulation in the same landscape type, the higher the value, the higher the patch cohesion.
	(DIVISION)	None	0 ≤ DIVISION < 1	Reflecting the degree of patch dispersion in the same landscape type. Value = 0, the landscape type is composed of a single patch; Value = 1, the landscape type is composed of many small patches.
Landscape Level	(CONTAG)	%	0<CONTAG ≤ 100	Describing the degree of agglomeration or extension of different landscape types.
	(SHDI)	None	0 ≤ SHDI	To characterize the complexity of the landscape as a whole, the greater its value, the higher the complexity of the landscape as a whole.

Note: The contents of the table are from the Fragstats 4.2 user manual.

According to the existing research, in addition to the impact of land use and landscape pattern, urban waterlogging disasters are also affected by rainfall and topographical factors. Therefore, the variables of rainfall, altitude, and land surface relief conditions were also considered.

Based on the daily rainfall data recorded at 50 meteorological stations, the daily rainfall distribution in the Shenzhen City area was obtained using the Ordinary Kriging (OK) method, and the average precipitation of each small watershed was extracted as research variables. Based on the original DEM data, the distribution of altitude and surface undulation in Shenzhen City were obtained, and the average value of each small watershed was then extracted as the research variable.

The above variables (Table 6) were acquired in ArcGIS 10.1 for further study.

**Table 6:** The classification and description of the independent variables.

Variable category	Variable subcategory		Variable names	Variable description
Landscape pattern index	4 rough classification	Green space, Built up, Water, Bare land	Built up_ Landscape pattern indexes	Under 4 rough classification circumstance, each Landscape pattern index has been calculated
			(LPI, COHESION, DIVISION)	
			4 classification_ Landscape pattern indexes	
			(CONTAG,SHDI)	
	16 fine classification	Arable land, Garden, Forest, Grassland, Business place, Warehousing and mining, Residential land,	Built up_ Landscape pattern indexes	Under 16 fine classification circumstance, each Landscape pattern index has been calculated
		Public land, Park,	(LPI, COHESION, DIVISION)	
		Special land, Traffic land, River, Other waters,	16 classification_ Landscape pattern indexes	
		Water facilities, wasteland,	(CONTAG,SHDI)	
		Equipment farmland,		
Other variables			Average daily rainfall, average elevation, average undulation	Statistics in small watersheds

### Methods

The dependent variable in the study was the waterlogging point density of each small watershed during the “5.11 rainstorm” in Shenzhen in 2014. The independent variables are shown in Table 3, which includes the landscape pattern index and other variables. A GWR was used to analyze the spatial non-stationarity of the influence of urban landscape pattern on urban waterlogging. The operation of this part was completed in GWR4.0.

#### GWR model

The traditional linear regression model only estimates parameters in an “average” or “global” way. If the independent variables are spatial data and there is a spatial autocorrelation between the independent variables, the traditional assumption of independent residuals in the regression model (ordinary least squares (OLS) model) cannot be satisfied. Therefore, the OLS model could not be applied in this study. The GWR model produces estimates for different regions to reflect the spatial non-stationarity of parameters in different spaces, and therefore the relationship between variables can be changed with a change of spatial position. The results are therefore more in line with objective reality. A GWR analysis was used in this study to extend the traditional regression framework and perform local parameter estimations based on the global regression model. The model structure is as follows:

1 $${y}_{i}={\beta }_{i0}({u}_{i},{v}_{i})+{\sum }_{k}{\beta }_{ik}({u}_{i},{v}_{i}){x}_{ik}+{\varepsilon }_{i}$$

where (*u*_*i*_, *v*_*i*_) is the geographic center coordinate of the *i* sample space unit, and *β*_*ik*_(*u*_*i*_*, v*_*i*_) is the value of the continuous function *β*_*ik*_(*u, v*) in the *i* sample space unit. If *β*_*ik*_ = *β*_*2k*_ = … =*β*_*nk*_, then the GWR model becomes a general linear model. Therefore, the spatial non-stationary nature of the data is the theoretical premise of establishing a GWR model.

Bandwidth b is a non-negative attenuation parameter between distance and weight^59^. The larger the bandwidth, the slower the weight changes with distance, and vice versa. When b is close to positive infinity, the weights of all observation points are close to 1, and the fitted value of the variable is close to the result of a general linear regression, making the model too smooth and causing excessive deviation. When the bandwidth tends to infinitesimal, the number of sample points participating in the regression calculation is too small, so that there is no influence between the parts, and the variance of the regression parameter estimation is too large, making the model too unsmooth. When b is constant, the weight of an observation infinitely far from the sample point *i* is close to zero. The determination of bandwidth is critical to the GWR model results, and therefore there are many ways to determine the optimal bandwidth^60,61^. This study uses the minimum Akaike information criterion (AIC) method^62,63^.

2 $${\rm{AIC}}=2n\,\mathrm{ln}(\sigma )+n\,\mathrm{ln}(2{\rm{\pi }})+n\left[\frac{n+tr({\rm{S}})}{n-2-tr({\rm{S}})}\right]$$

This equation is the maximum likelihood estimate of the random error variance, namely *σ* = RSS/n − tr(*S*), where tr(*S*) is the trace of the projection matrix *S* for the GWR model, which is related to b. For sample data, the minimum bandwidth for the AIC is the optimal bandwidth for the GWR model.

#### Autocorrelation analysis

Before the GWR model was established, a spatial autocorrelation analysis was performed on landscape features to verify whether they had spatial heterogeneity and spatial agglomeration characteristics^64^. A global spatial autocorrelation emphasizes the spatial dependence or spatial heterogeneity of the elements in the overall range, which is expressed as Moran’s *I* index. This index can be understood as the correlation coefficient between the factor observation and its spatial lag. The value of the index ranges from −1 to +1 and is calculated as follows:

3 $$I=\frac{n}{{\sum }_{i=1}^{n}{({X}_{i}-\bar{X})}^{2}}\frac{\mathop{\sum }\limits_{i=1}^{n}\mathop{\sum }\limits_{j=1}^{n}{w}_{ij}({X}_{i}-\bar{X})({X}_{j}-\bar{X})}{\mathop{\sum }\limits_{i=1}^{n}\mathop{\sum }\limits_{j=1}^{n}{w}_{ij}}$$

where *X*_*i*_ and *X*_*j*_ are the values of element *i* and *j, respectively; n* is the number of elements; *w*_*ij*_ is the spatial weight between elements *i* and *j*, which is defined as the reciprocal of the distance between them (if features are spatially neighbored, the value is 1, otherwise the value is 0).

The Z test value of formula (4) is:

4 $${\rm{Z}}(I)=\frac{I=E(I)}{\sqrt{V\,a\,r(I)}}$$

where *E*(*I*) is the mathematical expectation under the assumption that space does not agglomerate and *Var*(*I*) is the variance number. When the Z(*I*) value is positive and significant, it indicates that there is a positive spatial autocorrelation in the region, namely a high-high or low-low agglomeration. When the Z(*I*) value is negative and significant, there is a negative spatial autocorrelation in the region, namely a high-low or low-high agglomeration. When the Z(*I*) value is 0, the observation value is independently and randomly distributed. The local spatial autocorrelation emphasizes the degree of significant correlation of features at the local scale, which refers to the degree of similarity between each unit of the local space and its neighborhood, reflecting the degree to which each local unit obeys the global general trend, and is represented by a local indicators of spatial association (LISA) map^65^.
